# Clinical correlates of a subset of anti-CENP-A antibodies cross-reacting with FOXE3p53-62 in systemic sclerosis

**DOI:** 10.1186/ar4249

**Published:** 2013-07-09

**Authors:** Federico Perosa, Elvira Favoino, Giovanna Cuomo, Liboria Digiglio, Franco Dammacco, Marcella Prete, Gabriele Valentini, Vito Racanelli

**Affiliations:** 1 Rheumatology Unit, Department of Internal Medicine (DIMO), University of Bari Medical School, Piazza G. Cesare 11, 70124 Bari, Italy; 2 Rheumatology Section, F. Magrassi - A. Lanzara Department of Clinical and Experimental Internal Medicine, Second University of Naples, via Pansini 5, 80131 Naples, Italy; 3 Internal Medicine Section, Department of Internal Medicine (DIMO), University of Bari Medical School, Piazza G. Cesare 11, 70124 Bari, Italy

**Keywords:** Systemic sclerosis, CENP-A, peptide, FOXE-3, disease activity index

## Abstract

**Introduction:**

In a subset of patients with limited cutaneous (lc) systemic sclerosis (SSc), anti-CENP-A antibodies (Ab) cross-react with a peptide (FOXE3p53-62) that presents striking homology with one of the two immunodominant epitopes of CENP-A (Ap17-30). We searched for clinical correlates of anti-FOXE3p53-62 Ab by measuring their levels along with those of Ab to Ap17-30 and to the second immunodominant epitope of CENP-A, namely Ap1-17.

**Methods:**

Serum samples were obtained from 121 patients with SSc, 46 patients with systemic lupus erythematosus (SLE) and 25 healthy blood donors (HBD). The reactivity of serum IgG to Ap1-17, Ap17-30 and FOXE3p53-62 was measured by ELISA. The corresponding anti-peptide Ab were affinity-purified from pooled SSc sera and used to establish standard curves for quantifying these Ab in patients and HBD. Receiver operating characteristics (ROC) analysis, comparing SSc patients who were positive for anti-CENP Ab (ACA+) to those who were negative, was used to find cut-off points for dichotomizing the anti-peptide Ab levels into positive and negative. Clinical records were reviewed to extract demographic data and information about organ involvement and disease activity.

**Results:**

Of 121 SSc sera, 75 were ACA+; 88.0% of these samples reacted with Ap1-17, 82.6% with Ap17-30 and 53.3% with FOXE3p53-62. Among the 46 ACA- SSc sera, 2.2% reacted with Ap1-17, 4.3% with Ap17-30 and 11% with FOXE3p53-62. The levels of these Ab were low in ACA-, SLE and HBD groups and not significantly different among them. When ACA+ SSc patients were divided into subgroups positive or negative for anti-FOXE3p53-62 Ab, the only variables that were significantly different between groups were the levels of anti-Ap17-30 Ab and disease activity index (DAI). There was a significant association between negativity for anti-FOXE3p53-62 Ab and active disease defined as either DAI ≥3 (Fisher exact test, *P *= 0.045) or less restrictive DAI≥2.5 (*P *= 0.009).

**Conclusions:**

ACA+-Anti-FOXE3p53-62+Ab identifies a subgroup of patients with lcSSc who are less likely to develop active disease. In lc SSc patients at presentation, anti-FOXE3p53-62+ can be a marker with prognostic significance.

## Introduction

Systemic sclerosis (SSc), one of the most disabling connective tissue diseases, causes progressive fibrosis of skin and internal organs with a heterogeneous spectrum of clinical manifestations [1,2]. Despite several efforts to define prognostic markers and develop effective therapies (reviewed in [3]), its etiology and pathogenesis are largely unknown. SSc remains the connective tissue disease with the highest case-specific mortality, with 55% survival at 10 years [4]. Three main pathogenetic events have been identified as responsible for causing the disease, namely the development of vasculopathies (the earliest and possibly the primary event), increased collagen synthesis, and autoimmunity, the latter being characterized at the humoral level by the presence of anti-nuclear antibodies (ANA) in up to 95% of patients [2,5-7]. One subset of ANA is directed against the family of centromere-associated proteins (CENPs). Different types of anti-CENP antibodies (ACA) have been identified (anti-CENP-A to -H and anti-CENP-O) [8-12] and those directed to CENP-A, -B and -C are the most represented in sera of SSc patients with ACA [1,9,13,14].

Studies on the clinical correlates of anti-CENP-A or -B antibodies have shown that they are mostly found in sera of patients with limited SSc (>80% of cases) than diffuse SSc (18% to 40%) [5,15,16]. In addition, among ACA-positive (ACA^+^) SSc patients, the prevalence of pulmonary hypertension (without pulmonary fibrosis) in the early phase of the disease (10% to 20%) is higher than in ACA-negative (ACA^-^) patients (<1%) [15,17]. However, CENPs (and the corresponding Ab) do not appear to have any role to explain these clinical correlations. In order to understand why and how anti-CENP-A Ab are generated, we recently investigated their fine specificity, that is, the amino acids recognized by this Ab population, by focusing on the CENP-A region comprised between amino acids 17 and 30 (Ap17-30) [18]. This region was selected because it represents, along with the region spanning residues 1 to 17 (Ap1-17), the immunodominant epitope of CENP-A [19]. In that study, we defined two overlapping anti-CENP-A17-30 Ab motifs and found that one of them (PTPxxGPxxR) was also present in human forkhead box protein E3, a transcription factor encoded by the gene *FOXE3*. This nuclear protein, which plays an important role in lens epithelial-to-mesenchymal transition, had not previously been associated with SSc. We also observed that the corresponding FOXE3-derived peptide (FOXE3p53-62) was recognized by serum samples from only three of eight patients with anti-CENP-A17-30 Ab [18]. Here, we analyze the occurrence of anti-FOXE3p53-62 reactivity in sera from a larger sample of 121 patients with SSc (and in patients with systemic lupus erythematosus, SLE) in order to identify any possible clinical correlates of anti-FOXE3p53-62 Ab.

## Methods

### SSc patients

This retrospective study considered data from 121 patients with SSc who were being treated at the Rheumatology Units of the Universities of Naples and Bari. The patients were diagnosed according to the preliminary American College of Rheumatology criteria for the classification of the disease [20]. At the presentation, blood was drawn and the patients underwent an extensive medical evaluation, which included a patient's self report (for the recording of age at diagnosis and at the time of evaluation, sex and ethnicity), a routine history report, physical examination, and laboratory tests, including the assessment of blood cell counts, erythrocyte sedimentation rate (ESR), total serum protein (by capillary electrophoresis), C-reactive protein (CRP), titers of ANA, ACA and anti-topoisomerase-I (anti-topo-I) Ab, and complement proteins (C3, C4). C3 and C4 levels were considered low when their values were below the range of normal values. Moreover, tests were performed for renal (serum creatinine and urea) function.

On the basis of the presence of ACA and anti-topo-I Ab in their sera, determined on a routine basis using the anti-CENP-B and anti-Scl70 ELISA kits (Orgentec Diagnostika GmbH, Germany), the patients were allocated to two groups: (i) ACA^+ ^patients (*n *= 75); and (ii) ACA^- ^patients (*n *= 46). The ACA^- ^group comprised 38 patients with anti-topo-I Ab and 8 without. No ACA^+ ^patient had anti-topo-I Ab. None of the SSc patients had an overlapping vasculitis.

Disease duration was determined from the onset of the first Raynaud's manifestation [15]. The subtype of SSc, either limited or diffuse, was determined according to LeRoy *et al*. [21]. Skin involvement was assessed using the modified Rodnan skin score (mRss) [22] whereby the degree of skin thickness is measured in 17 areas and scored from 0 (normal skin) to 3 (severe thickening), for a total score range of 0 to 51. Bibasilar fibrosis for interstitial lung disease (ILD) was assessed with high-resolution computed tomography [23]. Regarding lung function, forced vital capacity (FVC) and diffusing lung capacity for carbon monoxide (DLCO) were measured and expressed as the percentage of predicted values. Systolic pulmonary arterial pressure (sPAP) was estimated from the tricuspid regurgitant jet velocity, measured using Doppler echocardiography. Pulmonary arterial hypertension (PAH) was defined as sPAP >35 mm Hg [24]. Clinical involvement of organs and tissues was assessed and scored (from 0 to 4) according to Medsger *et al*. [25] in the following domains: general, peripheral vascular, skin, joint/tendon, muscle, gastrointestinal tract, lung, heart, and kidney. Scores for the nine domains were totaled to obtain a disease severity score [25]. Moreover, the European Scleroderma Study Group (EScSG) disease activity index was calculated [26,27]. This index includes ten weighted items of which three are indicated by patients in reference to a possible change in conditions over the preceding month, and seven are clinical variables recorded by the physician. The former include skin deterioration (score, 0.0 or 2.0), vascular deterioration (0.0 or 0.5), and deterioration in heart/lung function (0.0 or 2.0); the latter include mRss>14 (score 0.0 or 1.0), scleredema (0.0 or 0.5), digital necrosis (0.0 or 0.5), arthritis (0.0 or 0.5), DLCO≤80% of predicted (0.0 or 0.5), ESR (first hour) >30 mm/h (0.0 or 1.5) and hypocomplementemia (C3, C4; 0.0 or 1.0). A disease activity index ≥3 was used to define the activity state of disease [27,28].

### Ethical issues

Approval for the collection of sera from patients and for the use of their clinical data for research purposes was obtained from the ethics committees of the University of Naples and University of Bari. All subjects provided written informed consent for the clinical samples and data to be used for research purposes.

### Serum samples, reagents and peptides

Serum samples from the 121 SSc patients and from 46 patients with SLE were obtained from the serum banks at the Departments of Internal Medicine (Universities of Bari and Naples). In addition, serum from 25 healthy blood donors (HBD) was obtained from the hospital blood bank of the University of Bari. SLE patients were used as a control group, because in both SSc and SLE the T helper 2 (Th2) immune response appears to play an important role in mediating the autoimmune damage [29,30]. Sera were stored at -80°C until use.

Electrophoresis reagents were purchased from Bio-Rad Laboratories (Segrate, Italy). Polyclonal human IgG preparations for intravenous use (IVIG, Intratec^®, ^Dreieich, Germany) were purchased from Biotest (Dreieich, Germany). Horseradish-peroxidase (HRP)-conjugated xeno-Ab to human IgG (Fc portion) was purchased from Jackson Immunoresearch Laboratories (Avondale, PA, USA). Unless otherwise specified, all other chemicals were purchased from BDH Merck (Poole, Dorset, UK) or Sigma-Aldrich (St Louis, MO, USA). Peptides were synthesized by Primm (Milan, Italy). Their purity, determined by analytical reverse phase chromatography and mass spectrometry, ranged between 91.3% and 100%. Peptides were coupled to BSA by means of glutaraldehyde, as previously described [31].

### Serological assays (indirect ELISA)

The reactivity and specificity of Ab with peptide were assessed by indirect ELISA, as described [32], with minor modifications. Briefly, U-bottom 96-well polyvinylchloride plates (BD Falcon, Franklin Lakes, NJ, USA) were incubated with 50 µl PBS containing 5 µg/ml BSA-conjugated peptide, for 12 h at 4°C. Wells were washed once with PBS containing 0.05% Tween 20 (PBS-T20) and blocked with PBS containing 0.5% BSA (PBS-BSA). Serum samples (diluted 100 times in PBS-BSA) were added to the wells and incubated for 4 h at 25°C. Wells were washed three times with PBS-T20. Bound IgG was detected by sequential incubation with HRP-conjugated xeno-Ab to the Fc portion of human IgG (1 h at 25°C) and o-phenylenediamine (0.5 mg/ml; 100 µl/well); color development was stopped by adding 100 µl 2 N H2SO4 and the absorbance was read at 490 nm with the Benchmark microplate reader (Bio-Rad Laboratories). Background binding was determined from the absorbance generated in wells with blocking solution alone. Specific binding was determined by subtracting the background absorbance from the absorbance in experimental wells. Samples with the highest binding for each peptide were selected for affinity purification of the corresponding Ab.

### Affinity purification of human autoantibodies

CENP-A-derived peptides Ap1-17 (1MGPRRRSRKPEAPRRRS17) and Ap17-30 (17SPSPTPTPGPSRRG30) and FOXE3-derived peptide FOXE3p53-62 (53PTPAPGPGRR62) were conjugated to Affi-Gel 15 (Bio-Rad Laboratories) at a concentration of 2 mg/ml resin following the manufacturer's instructions; these conjugated resins were used to affinity-purify the corresponding anti-peptide Ab as previously described [18], from a pool of a few serum samples with the highest binding avidity. Briefly, 5 ml pooled serum was diluted in an equal volume of PBS and repeatedly passed through a BSA-conjugated Affi-Gel 15 column, and then absorbed several times on a peptide-conjugated Affi-Gel 15 column. Bound IgG was eluted, dialyzed overnight against PBS and concentrated by lyophilization. Ab concentration was determined by UV absorption with a 1.35 extinction coefficient at 280 nm for 1 mg/ml protein. The extent of contamination of samples by human serum albumin was assessed by SDS-PAGE, Coomassie brilliant blue staining, and density scanning; the final concentration of IgG was corrected to reflect this contamination. The specificity of purified Ab for peptides was assessed as previously described [18].

### ELISA quantification of autoantibody levels in sera

Serum levels of anti-Ap1-17, anti-Ap17-30 and anti-FOXE3p53-62 IgG were measured by ELISA as previously described [32]. Briefly, 96-well polyvinylchloride plates were coated with 50 µl PBS containing 5 µg/ml BSA-conjugated peptide, for 12 h at 4°C. Wells incubated with BSA alone were used as negative controls. Wells were washed once with PBS-T20 and blocked with PBS-BSA. Standard (calibration) curves were generated by incubating the wells with known concentrations of affinity-purified anti-peptide Ab up to a maximum 2.5 µg/ml. To quantify the concentration of anti-peptide Ab in serum samples, 50 µl of 4-fold serial dilutions of sera (starting dilution 1:40) was added to the wells. After 4 h, wells were washed three times with PBS-T20. The assay was then continued as described above (Serological assays). Peptide-specific IgG concentration (µg/ml) was determined from the calibration curve and corrected for the dilution factor.

### Statistical analyses

Receiver operating characteristics (ROC) analysis was used to find cutoff points for dichotomizing continuous variables [33]. This analysis was performed using MedCalc software (v. 7.6.0.0) (MedCalc Software bvba, Ostend, Belgium), and the discriminating cutoff was automatically obtained. The Mann-Whitney test was used with continuous variables for comparisons between groups, the chi-squared and Fisher exact tests were used to define associations among dichotomized variables, and multivariate analysis was used to define independent associations between two variables; these statistical tests were performed using SPSS v. 20 for Windows. A *P*-value <0.05 indicated statistical significance.

## Results

The 75 ACA^+ ^and 46 ACA^- ^patients (Table 1) were similar in sex distribution, age at diagnosis and disease duration. ACA^+ ^patients were more likely to have the limited subtype of SSc (93.3% versus 52.2%; *P *<0.0001), and as a group had lower mRss values for skin involvement (mean 3.9 versus 9.0; *P *<0.0001). Despite similar values of sPAP and rates of PAH, the ACA^+ ^group had a lower frequency of ILD (18.0% versus 78.6%; *P *<0.0001) and higher percentages of predicted FVC and DLCO. Their overall disease severity score was also lower (mean 5.17 versus 7.20; *P *= 0.009) and this was reflected in significant differences between groups in the two domains of skin and joint/tendon involvement. No significant difference was observed for disease activity index.

**Table 1:** Clinical characteristics of 121 patients with systemic sclerosis (SSc) according to the presence or absence of anti-centromere-associated protein antibodies (ACA)

Variable	All patients	ACA-positive (*n *= 75)	ACA-negative (*n *= 46)	*P*-value
Female, n (%)	113 (93.4)	71 (94.7)	42 (91.3)	0.470^a^
Age at diagnosis, years	38.8 (14.3)	39.3 (13.7)	37.8 (15.5)	0.530^b^
Disease duration, years	17.1 (10.3)	18.4 (10.6)	14.7 (9.4)	0.050^b^
Limited disease, n (%)	94 (77.7)	70 (93.3)	24 (52.2)	<0.0001 ^a^
mRss	5.70 (7.00)	3.90 (6.40)	9.00 (7.10)	<0.0001 ^b^
FVC, % of predicted	105.60 (20.20)	107.40 (19.10)	84.30 (22.20)	0.014^b^
DLCO, % of predicted	73.10 (23.80)	76.60 (25.00)	64.10 (17.80)	0.017^b^
sPAP, mm Hg	28.10 (10.60)	27.70 (11.60)	28.80 (8.80)	0.058^b^
ILD, n (%)	24 (27.6)	13 (18.0)	36 (78.6)	<0.0001^a^
PAH, n (%)	16 (13.2)	8 (10.7)	8 (17.4)	0.289^a^
Disease severity scale				
Total score	5.89 (3.46)	5.17 (2.80)	7.20 (4.12)	0.009^b^
General	0.50 (0.80)	0.44 (0.70)	0.60 (0.93)	0.484^b^
Peripheral vascular	1.52 (0.71)	1.46 (0.68)	1.61 (0.76)	0.212^b^
Skin	0.83 (0.65)	0.66 (0.64)	1.14 (0.56)	<0.0001^b^
Joint/tendon	0.47 (1.00)	0.26 (0.64)	0.85 (1.35)	0.012^b^
Muscle	0.16 (0.50)	0.10 (0.42)	0.26 (0.62)	0.090^b^
Gastrointestinal tract	0.83 (0.70)	0.77 (0.66)	0.94 (0.72)	0.200^b^
Lung	1.34 (1.11)	1.22 (1.18)	1.54 (0.96)	0.111^b^
Heart	0.24 (0.50)	0.24 (0.42)	0.25 (0.63)	0.514^b^
Kidney	0.05 (0.41)	0.0 (0.0)	0.14 (0.68)	0.058^b^
Disease activity index	1.52 (1.68)	1.26 (1.31)	1.98 (2.14)	0.128^b^

Values are presented as mean (SD) unless otherwise indicated. ^a^Chi-squared test; ^b^Mann-Whitney *U*-test. mRss, modified Rodnan skin score; FVC, forced vital capacity; DLCO, diffusing lung capacity for carbon monoxide; sPAP, systolic pulmonary arterial pressure; PAH, pulmonary arterial hypertension (sPAP >35 mm Hg); ILD, interstitial lung disease.

The study included two additional control groups: 46 patients with SLE and 25 HBD. The mean (± SD) age at diagnosis of the SSc patients (38.8 ± 14.3) was similar to that of the SLE patients (34.5 ± 9.6) and HBD (34.5 ± 4.6). As expected, among both SSc and SLE patients, the female sex predominated (94.7% and 91.3%, respectively), but there was a more balanced sex distribution among HBD (56% female). All patients and HBD were self-declared white Caucasians.

### Levels of anti-Ap1-17, anti-Ap17-30 and anti-FOXE3p53-62 Ab in patients' sera

To quantify anti-peptide Ab in the patients' sera, we first purified these antibodies from samples with the highest binding avidity to generate standard curves in indirect ELISA. Based on an initial ELISA screening of the 75 ACA+ sera with Ap1-17, Ap17-30 and FOXE3p53-62 peptides, sera from three, four and three patients, respectively, were pooled and used in affinity chromatography; this resulted in the purification of anti-Ap1-17 and anti-Ap17-30 Ab, but not anti-FOXE3p53-62 Ab. The calibration curves for anti-Ap1-17 and anti-Ap17-30 Ab permitted detection in the range of 0.28 to 6037 µg/ml, and 0.08 to 32.85 µg/ml, respectively. Because attempts to purify Ab from a FOXE3p53-62-conjugated column were unsuccessful, the calibration curve to measure these serum levels was constructed using anti-Ap17-30-specific IgG purified from a pool of the three additional SSc serum samples displaying the highest binding to FOXE3p53-62. The range of detection of this assay was 1.32 to 880 µg/ml.

**Figure 1:**
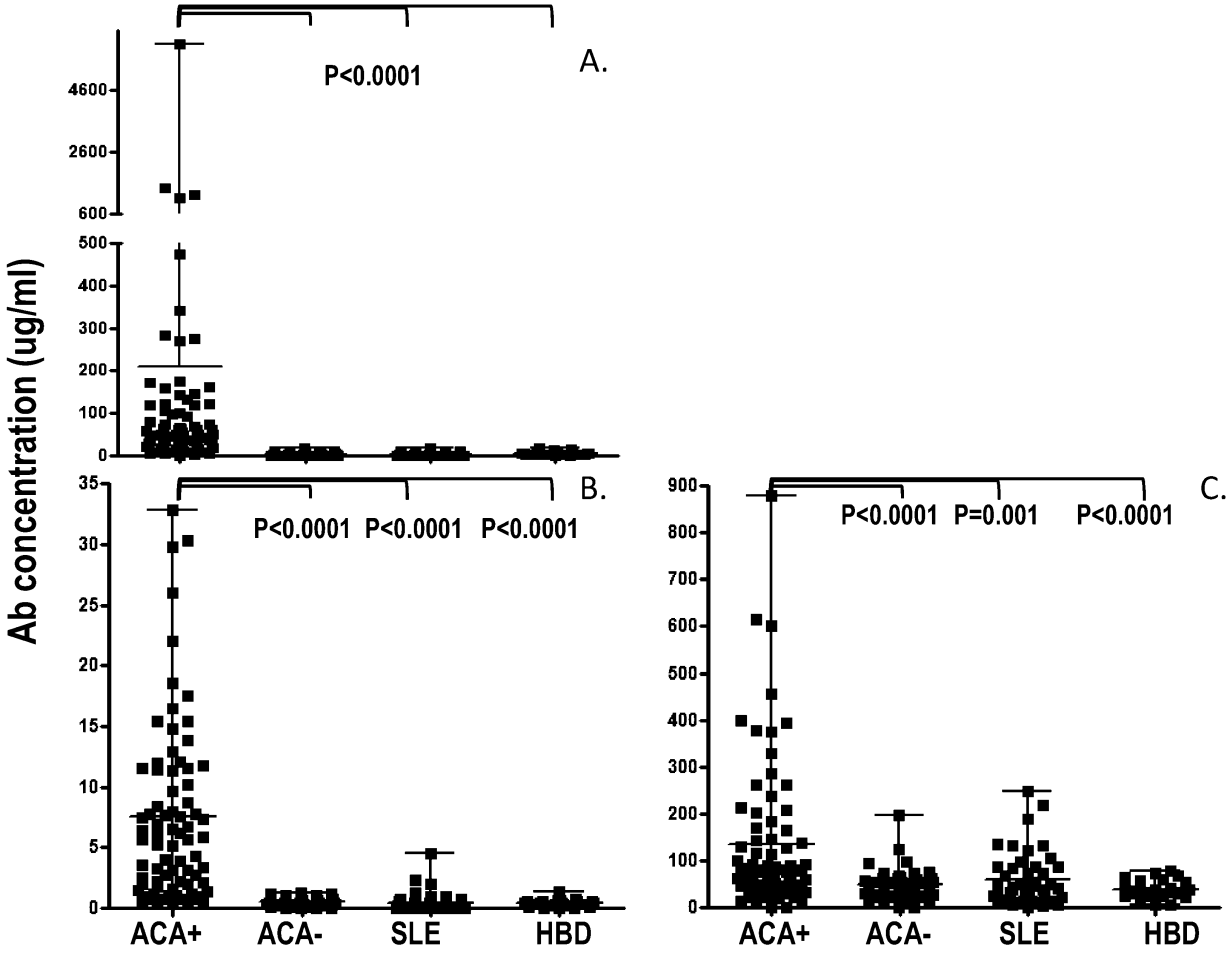
**Levels (mean, range) of antibodies to anti-Ap1-17 (panel A), -Ap17-30 (panel B) and anti-FOXE3p53-62 (panel C) in patients with systemic sclerosis (anticentromere antibody-positive and -negative) and systemic lupus erythematosus, and in healthy blood donors**. Ab levels were measured by enzyme-linked immunosorbent assay (ELISA), using appropriate standard curves. The proportion of the difference of the levels between groups was evaluated by the Mann-Whitney *U*-test. *P *<0.05 was considered significant. Ab, autoantibodies; ACA+, anticentromere antibody-positive; ACA-, anticentromere antibody-negative; SLE, systemic lupus erythematosus; HBD, healthy blood donors.

The levels of anti-Ap1-17, -Ap17-30 and -FOXE3p53-62 IgG in sera of 75 ACA^+ ^SSc patients, 46 ACA^- ^SSc patients, 46 SLE patients and 25 HBD are illustrated in Figure 1. As expected, the levels of anti-Ap1-17 and -Ap17-30 IgG in ACA^+ ^SSc patients were markedly higher than in the other groups (Mann-Whitney, P <0.0001); the mean levels of these Ab in the other groups were at least 10-fold lower and not significantly different among groups. Anti-FOXE3p53-62 Ab were again highest in the ACA^+ ^SSc group (*P *<0.0001 versus ACA^- ^SSC; *P *= 0.001 versus SLE patients; *P *<0.0001 versus HBD). These data suggest that anti-FOXE353-62 reactivity is more pronounced in ACA^+ ^SSc patients.

### Dichotomization of anti-peptide Ab levels in relation to the presence or absence of ACA

**Figure 2:**
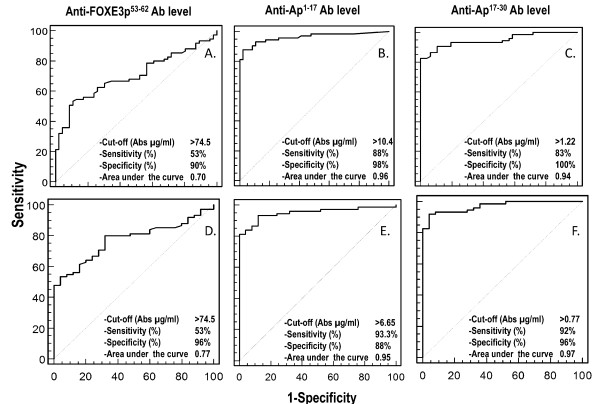
**Receiver operating characteristic analysis to define the best cutoffs of anti-peptide (Ap1-17, Ap17-30 and FOXE3p53-62) autoantibody levels discriminating anticentromere antibody-positive from and anticentromere antibody-negative patients (panels A to C), or from healthy blood donors (panels D to F)**. Ab, autoantibodies.

As a first step to identifying clinical correlates of anti-FOXE353-62 Ab, we used ROC analysis to define cutoff concentrations of the three anti-peptide Ab that could discriminate between ACA^+ ^and ACA^- ^patients. When the analysis was performed for anti-FOXE3p53-62 IgG, the concentration that best discriminated the ACA^+ ^and ACA^- ^groups was 74.5 µg/ml (sensitivity, 53%; specificity, 90%; area under the curve (AUC), 0.70) (Figure 2A). If instead of the ACA^- ^group we used HBD as the control, we obtained the same cutoff and sensitivity (Figure 2D) and slightly higher specificity (96%) and AUC (0.77). When ROC analysis was performed for anti-Ap1-17 IgG using both ACA^- ^(Figure 2,B) and HBD (Figure 2E) as the comparison groups, the sensitivity (88% and 93%, respectively), specificity (98% and 88%) and AUC (0.96 and 0.95) were markedly higher, as expected by the fact that Ap1-17 and Ap17-30 are CENP-A-derived peptides. Similar results were obtained for anti-Ap17-30 IgG, with sensitivity (83% and 92%, respectively), specificity (100% and 96%) and AUC (0.94 and 0.97) versus ACA^- ^(Figure 2C) and HBD (Figure 2F).

Using the three cutoffs for ACA^+ ^versus ACA-, we dichotomized serum levels of anti-Ap1-17, -Ap17-30 and -FOXE3p53-62 Ab as positive (above the cutoff) or negative (below the cutoff). Among ACA^+ ^SSc patients, three were negative for all three Ab, thirty-five were positive for all three Ab, and thirty-seven were positive for one or two of the Ab (the most common combination was anti-Ap1-17 and anti-Ap17-30 in twenty-one patients). Comparing across groups (Table 2), anti-Ap1-17 Ab and anti-Ap17-30 Ab were scored as being positive in 66 and 62 ACA^+ ^SSc patients (88.0% and 82.6%, respectively), whereas low frequencies were observed in the other groups. Anti-FOXE3p53-62 Ab were scored positive in 40 ACA^+ ^SSc patients (53.3%) and in 13 (28.3%) SLE patients. Looking further at the concomitant expression of the different Ab, all 40 ACA^+ ^SSc patients who were positive for anti-FOXE3p53-62 Ab were also positive for anti-Ap1-17 Ab or anti-Ap17-30 Ab (or both). In contrast, only three (23.1%) of the thirteen SLE patients who were positive for anti-FOXE3p53-62 Ab were positive for at least one of the other Ab. These data suggest that the expression of Ab to FOXE3p53-62 and to Ap1-17 or Ap17-30 may be associated in SSc but not in SLE.

**Table 2:** Presence of anti-peptide IgG in sera from patients with SSc or SLE and healthy blood donors, according to the best cutoffs for discriminating ACA-positive from ACA-negative SSc patients

IgG specificity	Discriminating	SSc		SLE (*n *=46)	HBD (*n *=25)
				
	cutoff (µg/ml)^a^	ACA^+^ (*n *= 75)	ACA^+^ (*n *=46)		
Ap1-17^+^	>10.4	66 (88.0)	1 (2.2)	2 (4.3)	3 (12.0)
Ap17-30^+^	>1.2	62 (82.6)	2 (4.3)	4 (8.7)	1 (4.0)
FOXE3p53-62^+^	>74.5	40 (53.3)	5 (10.9)	13 (28.3)	1 (4.0)
FOXE3p53-62^+ ^and Ap1-17^+ ^or Ap17-30^+^		40 (100)^b^	0 (0) ^b^	3 (23.1) ^b^	1 (100) ^b^

Values are presented as n (%) from a sample of 121 patients with SSc, 46 patients with SLE, and 25 HBD. aCutoffs were defined using receiver operating characteristic analysis; bpercentage of FOXE3p53-62+ samples that were also positive for Ap1-17 and/or Ap17-30. ACA, anti-centromere-associated protein antibodies; SSc, systemic sclerosis; SLE, systemic lupus erythematosus; HBD, healthy blood donors.

### Clinical correlates of anti-FOXE3p53-62 Ab

To identify possible clinical correlates of anti-FOXE3p53-62 Ab, the ACA^+ ^SSc patients were divided into subgroups according to whether they scored positive (*n *= 40) or negative (*n *= 35) (Table 3). The subgroups were similar for most clinical and laboratory variables, including the levels of anti-Ap1-17 Ab. There were significant differences, however, in the levels of anti-Ap17-30 Ab, which were higher in the FOXE3p53-62-positive group (Mann-Whitney *P *<0.0001), and in the disease activity index, which was higher (more active disease) in the FOXE3p53-62-negative group (Mann-Whitney *P *= 0.038). The difference in the disease activity index was explained by higher scores in two particular domains, namely deterioration in heart/lung function and hypocomplementemia.

**Table 3:** Clinical characteristics of 75 ACA^+ ^(>74

Variable	Anti-FOXE3p53-62		*P*-value
		
	Positive (*n *= 40)	Negative (*n *= 35)	
Female, n (%)	38 (95.0)	33 (94.3)	1.000^a^
Age at diagnosis, years	37.4 (14.2)	41.5 (13.0)	0.217^b^
Disease duration, years	20.3 (11.9)	16.2 (8.5)	0.151^b^
Disease subtype, limited, n (%)	37 (92.5)	33 (94.3)	0.757^a^
Anti-Ap1-17 IgG, µg/ml	252.50 (954.00)	161.00 (313.00)	0.807^b^
Anti-Ap17-30 IgG, µg/ml	9.63 (7.00)	5.23 (7.20)	<0.0001^b^
mRss	3.70 (4.20)	4.10 (8.40)	0.533^b^
FVC, % of predicted	110.6 (17.0)	103.7 (20.6)	0.201^b^
DLCO, % of predicted	76.2 (24.0)	76.6 (24.6)	0.727^b^
sPAP, mm Hg	29.00 (14.20)	26.20 (8.20)	0.510^b^
ILD, n (%)	8 (20.0)	5 (15.2)	0.590 ^a^
PAH, n (%)	5 (12.5)	3 (8.6)	0.719^a^
Disease severity score	5.30 (2.70)	5.00 (2.30)	0.570^b^
Disease activity index	0.91 (0.91)	1.61 (1.61)	0.038^b^
Disease activity index domains			
mRss >14 (0.0, 1.0)	0.02 (0.16)	0.03 (0.17)	0.980
Scleredema (0.0, 0.5)	0.11 (0.21)	0.11 (0.21)	0.971
Skin (0.0, 2.0)	0.05 (0.30)	0.10 (0.50)	0.481
Digital necrosis (0.0, 0.5)	0.07 (0.18)	0.04 (0.14)	0.396
Vascular (0.0, 0.5)	0.06 (0.16)	0.06 (0.16)	0.887
Arthritis (0.0, 0.5)	0.07 (0.20)	0.06 (0.16)	0.652
DLCO <80% of predicted (0.0, 0.5)	0.26 (0.25)	0.20 (0.25)	0.282
Heart/lung function (0.0, 2.0)	0.05 (0.31)	0.40 (0.81)	0.015
ESR >30 mm/h (0.0, 1.5)	0.18 (0.50)	0.30 (0.60)	0.380
Low C3 or C4 (0.0, 1.0)	0.05 (0.22)	0.31 (0.50)	0.003

Values are presented as mean (SD) unless otherwise indicated. ^a^Chi-squared test; ^b^Mann-Whitney *U*-test; a *P*-value <0.05 was considered significant. SSc, systemic sclerosis; ACA, anti-centromere-associated protein antibodies; mRss, modified Rodnan skin score; FVC, forced vital capacity; DLCO, diffusing lung capacity for carbon monoxide; sPAP, systolic pulmonary arterial pressure; ILD, interstitial lung disease assessed by high resolution computed tomography; PAH, pulmonary arterial hypertension (sPAP >35 mm Hg); ESR, erythrocyte sedimentation rate in the first hour. Cardiac involvement was evaluated by electrocardiography, echocardiography and physical examination.

**Figure 3:**
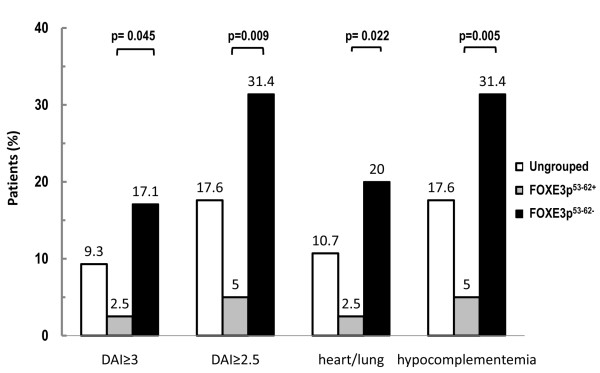
**Frequency of active disease in anticentromere antibody-positive systemic sclerosis patients, according to the positivity/negativity of anti-FOXE3p53-62 autoantibodies**. Patients were divided into anti-FOXE3p53-62-positive and -negative groups according to the cutoff (>74.5 µg/ml) that best discriminated **anticentromere antibody (ACA)-positive **from ACA-negative patients with systemic sclerosis. Active disease was defined as a disease activity index (DAI) ≥3.0 or ≥2.5. Patients were scored as being positive or negative for the 10 items of the DAI. Differences between groups were assessed using Fisher's exact text. No significant differences were observed in the remaining eight items of the DAI.

This clinical association was explored further by looking at the numbers of ACA^+ ^patients with active disease (defined as a disease activity index ≥2.5 or ≥3.0) and by analyzing the disease activity data as frequencies of patients with particular symptoms in each domain (Figure 3). The FOXE3p53-62-negative group had higher percentages of patients with active disease when defined both with a cutoff of 3.0 (Fisher exact test, *P *= 0.045) and a less restrictive criterion of 2.5 (*P *= 0.009). For the individual index items, the FOXE3p53-62-negative group had higher percentages of patients with deterioration in heart/lung function and hypocomplementemia; no other significant differences between groups were detected for the remaining items (data not shown).

Finally, multivariate regression was performed to detect any independent associations between the levels of anti-FOXE3p53-62 Ab in ACA^+ ^SSc patients and clinical variables and to identify any possible confounding factors such as age, disease duration and ANA titer (Table 4). The level of anti-Ap17-30 Ab and disease activity index were retained in the model as the only independent variables. Disease activity index displayed an inverse association with anti-FOXE3p53-62 Ab levels: positivity for anti-FOXE3p53-62 Ab was associated with low scores of the disease activity index after statistical correction for multiple comparisons (*P *= 0.035). When multivariate regression analysis was performed including complement as a dichotomized variable, complement (B = -2.42; Exp(B) = 0.089; *P *= 0.030) and anti-Ap17-30 Ab level (B = 0.115; Exp(B) = 1.12; *P *= 0.028) were retained as the only independent variables. Altogether, these data indicate that ACA^+ ^SSc patients with high anti-FOXE3p53-62 Ab levels are significantly less susceptible to developing active disease than ACA^+ ^SSc patients with low levels of these Ab.

**Table 4:** Logistic multivariate regression to detect independent associations between anti-FOXE3p53-62 Ab levels and clinical and laboratory parameters (continuous and dichotomized) in 75 ACA^+ ^patients

Variable	B coefficient	Exp (B)	*P*-value^a^	95% CI
Gender	-0.065	1.067	0.956	0.104, 0.914
Age at diagnosis	-0.022	0.978	0.465	0.922, 1.038
Disease duration	0.039	1.040	0.661	0.943, 1.096
mRss	-0.06	0.942	0.277	0.846, 1.049
ESR	0.004	1.004	0.847	0.962, 1.049
CRP	-0.052	0.949	0.749	0.689, 1.307
ANA titer	0.000	1.000	0.550	0.999, 1.002
Anti-Ap1-17 IgG	0.00	1.000	0.678	0.999, 1.001
Anti-Ap17-30 IgG	0.108	1.114	0.035	1.007, 1.231
Disease severity score	0.138	1.148	0.315	0.977, 1.501
Disease activity index	-0.711	0.491	0.035	0.254, 0.950

^a^A *P*-value >0.05 was not considered statistically significant. ACA, anti-centromere-associated protein antibodies; ANA, anti-nuclear antibodies; CRP, C-reactive protein; ESR, erythrocyte sedimentation rate; mRss, modified Rodnan skin score.

## Discussion

In a previous study, we defined the fine specificity of anti-CENP-A Ab and discovered that one of the two identified binding motifs was present not only in CENP-A but also in FOXE3, a protein not previously implicated in SSc [18]. As a first step to understanding if anti-FOXE3 Ab are of clinical interest, we measured their levels in patients with SSc and SLE and in HBD and searched for clinical correlates. This work revealed that SSc patients who are positive for anti-FOXE3p53-62 Ab are less likely to have active disease than SSc patients who have ACA but are negative for anti-FOXE3p53-62.

SSc patients who tested positive for ACA on standard laboratory tests were frequently found to have Ab reactivity to CENP-A peptides: 88.0% and 82.2% of sera from ACA^+ ^SSc patients reacted with Ap1-17 and Ap17-30, respectively. This finding parallels a similar observation by Akbarali *et al*. [19], who found that 80% of ACA^+ ^sera reacted with the CENP-A peptide spanning amino acids 17 to 30. It is likely that the higher percentage of reactivity recorded in the present study reflects the different methodology employed, because Akbarali et al. used peptide-coated pins, whereas we used BSA-conjugated peptide adsorbed onto microtiter plate wells. These findings, together with the high specificity and sensitivity determined by ROC analysis, suggest that Ap17-30 and Ap1-17 peptides can be used as antigens in ELISAs just as effectively as recombinant CENPs currently used in commercial ACA assays.

The frequencies of different disease manifestations in ACA^+ ^and ACA- SSc patients are similar, though not identical to those reported in a recent study by Hanke *et al*. [34]. In both studies, skin involvement and ILD was more frequent in the ACA^- ^group. At variance from Hank *et al*., however, we found no significant differences regarding cardiac involvement. The discrepancy in the results may reflect the different percentages of ACA^- ^patients with limited disease recorded in two studies, which was 18.4 % in the cohort of Hanke et al., whereas it was 52.2% in our study.

The apparent discrepancy between heart/lung function (significantly different between anti-FOXE3p53-62-positive and -negative groups) on one hand, and FVC, DLCO, sPAP, ILD and PAH (equally distributed between groups) on the other, is likely to reflect the differences in the significance of heart/lung function compared to the other functions. In fact, while the former is based on the patients' self-assessments of the deterioration of cardiopulmonary manifestations (mainly based on the severity of effort dyspnea, which occurred in the previous month), FVC, DLCO, sPAP, ILD and PAH record the degree of functional deterioration that cannot be strictly correlated to symptoms. Supporting this possibility was the lack of any significant association (chi-squared test) between the heart/lung item of the disease activity index and FVC (<80%), DLCO (<80%) or sPAH (>35 mm Hg) (data not shown).

The biological significance of ACA reactivity with FOXE3p53-62 deserves some comments. One can argue that due to the homology of FOXE3p53-62 and CENP-A-derived Ap17-30 (80% identical, 10% strongly similar) [18], anti-FOXE3p53-62 Ab are simply CENP-A17-30-specific Ab that cross-react with FOXE3p53-62. Though this possibility cannot be ruled out in ACA^+ ^patients, considering the positive association between levels of anti-FOXE3p53-62 and anti-Ap17-30 Ab, two lines of evidence suggest that anti-FOXE3p53-62 Ab can be generated independently of anti-Ap17-30. First, 46.7% of sera from the ACA^+ ^group reacted with Ap17-30 peptide only, indicating that in certain patients, anti-CENP-A Ab do not necessarily cross-react with FOXE3p53-62. Second, sera from two ACA^- ^SSc patients and ten SLE patients reacted with FOXE3p53-62 only. Although the significance of the presence of FOXE3p53-62-specific Ab in SLE is unknown, it is clear that under certain conditions the expression of anti-FOXE3p53-62 Ab is dissociated from that of anti-CENP-A Ab.

A significantly lower disease activity index was recorded in the subset of ACA^+ ^SSc patients who were FOXE3p53-62-positive. Although this subgroup was relatively small, as were the numbers of patients with active disease, this finding was confirmed by reanalysis using the Fisher test and by multivariate analysis, which excluded any influence of potential confounding factors (patient's age, disease duration, ANA titer, or ESR). Multivariate analysis also highlighted the independent associations between active disease and low levels of anti-FOXE3p53-62 Ab and indicated that a low complement-domain of the disease activity index plays a major role in the association.

The clinical correlation of disease severity scores and auto-Ab has been extensively reported in SSc (reviewed in [16,35,36]). Examples include a higher incidence of: 1) lung involvement in anti-topo-I- [37,38] and anti-Ro52/TRIM21-positive patients [39]; 2) renal crisis and poorer prognosis in anti-RNA polymerase (RNAP)I/III-positive patients [40,41]; 3) PAH in patients with anti-vascular smooth muscle cell Ab [42] or in ACA^+ ^patients [15,37,43], and 4) digital ulceration and muscular involvement in anti-PM/Scl-75/100-positive patients [44] and in anti-U3RNP Ab-positive patients [45]. Even so, none of these investigations have shown any correlation between SSc auto-Ab and disease activity. To the best of our knowledge, this is the first report to describe the ability of auto-Ab to discriminate a subset of ACA^+ ^patients, namely those who are less likely to have active disease.

It is not clear whether high levels of anti-FOXE3p53-62 Ab protect patients from developing active disease or, on the contrary, whether the lack of an active disease state promotes the generation of anti-FOXE3p53-62 Ab. We favor the first possibility, as preliminary results on serial samples of six patients collected over a period of 2 years suggest that the levels of these Ab remain constant. Moreover, as this was a two-center study, additional studies are needed to validate our findings in patients being cared for at multiple institutions.

## Conclusions

The measurement of anti-FOXE3p53-62 Ab levels adds specific clinical information beyond that provided by ACA measurements, by defining a subgroup of SSc patients (FOXE3p53-62-positive) who are less likely to develop active disease. Future follow-up studies will evaluate how the presence of these Ab influences the prognosis of these patients.

## Abbreviations

Ab: antibody/antibodies; ACA: anti-centromere antibody; ANA: anti-nuclear antibody; anti-topo-I: anti-topoisomerase-I; AUC: area under the curve; BSA: bovine serum albumin; C3: complement protein 3; CRP: C-reactive protein; DAI: disease activity index; DLCO: diffusing lung capacity for carbon monoxide; ELISA: enzyme-linked immunosorbent assay; EScSG: European Scleroderma Study Group; ESR: erythrocyte sedimentation rate; FOXE3: forkhead box E3; FVC: forced vital capacity; HBD: healthy blood donors; HRP: horseradish peroxidase; Ig: immunoglobulin/s; ILD: interstitial lung disease; IVIG: polyclonal human immunoglobulins G for intravenous use; lc: limited cutaneous; mRss: modified Rodnan skin score; PAH: pulmonary arterial hypertension; PBS: phosphate-buffered saline; PBS-T20: phosphate-buffered saline containing 0.05% Tween 20; ROC: receiver operating characteristics; SLE: systemic lupus erythematosus; sPAP: systolic pulmonary arterial pressure; SSc: systemic sclerosis; Th: T helper.

## Competing interests

The authors declare that they have no competing interests.

## Authors' contributions

FP, EF, and FD conceived and designed the study; FP and EF drafted the manuscript. GV and FD participated in critically revising the manuscript for important intellectual content. GV, GC, VR, and MP undertook recruitment of patients and collection of clinical data. EF and LD carried out the immunoassay and acquired the data. EF, LD, and GC participated in the analysis and interpretation of data. FP, VR, and MP performed statistical analysis. All authors read, revised and approved the final version of the manuscript.

## Ackowledgements

The authors are grateful to Mr Vito Iacovizzi for his excellent secretarial assistance. Valerie Matarese provided scientific editing.
